# Reply: Prediction remains difficult—in particular when it involves the future!

**DOI:** 10.1016/j.xjon.2021.10.002

**Published:** 2021-10-13

**Authors:** Christian Stoppe

**Affiliations:** Department of Anesthesiology and Intensive Care Medicine, University Hospital Würzburg, Würzburg, Germany

Reply to the Editor:

**Figure:**
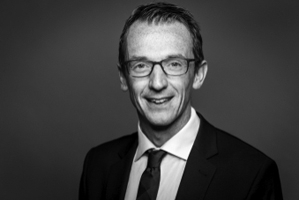

The author reported no conflicts of interest.

The *Journal* policy requires editors and reviewers to disclose conflicts of interest and to decline handling or reviewing manuscripts for which they may have a conflict of interest. The editors and reviewers of this article have no conflicts of interest.

Over the past few decades, a wide variety of risk-stratification systems have been investigated and developed to quantify the perioperative risk of patients who undergo cardiac surgery.^1^^,^^2^ While initial attempts focused on mortality alone, more recent models have been proposed, allowing for the prediction of postoperative complications such as renal failure, prolonged ventilation, infectious complications, neurologic deficits, and the detection of functional recovery. More recent risk-stratification models have been developed from large databases of patients undergoing cardiac surgery using preoperative patient and surgical factors to assess their predictive value for postoperative complications.^3^ We agree with the comments given by Dr Carosella that actual risk scores should be considered as adjunctive to further improve the clinical judgment and not replace it.^4^ Furthermore, several other factors beyond the nutritional risk alone are of relevance for an adequate assessment of the patient's perioperative risk, including age and frailty, among others. Cho and colleagues^5^ previously demonstrated in an observational, single-center retrospective study that the nutritional status of patients who undergo cardiac surgery may significantly influence long-term survival after valve surgery and thus should receive more recognition. However, based on the limitations of the study design, we agree with Dr Carosella that the received findings should be cautiously considered as purely hypothesis-generating. Furthermore, it is important to note that the developed model did not derive from large data sets as Society of Thoracic Surgeons or EuroSCORE (European System for Cardiac Operative Risk Evaluation) were. Yet, it is important to note that exploratory studies, such as those provided by Cho and colleagues,^5^ are needed to further develop new risk-stratification strategies and identify potential new factors, such as the nutritional risk assessment, which has remained under-recognized for a long time in this patient population.^6^^,^^7^ Therefore, studies like these can provide new perspectives, which may improve currently used risk scores. Beyond just the nutritional risk, other factors as named by Dr Carosella, to include frailty, mobility, cognitive status, or activities of daily living, are of clinical relevance and increasingly considered to be used for preoperative risk assessment. Yet, a prospective validation of any clinical meaningful characteristics should always be considered before their use in risk scores, as previous studies highlighted that potentially relevant risks such as frailty failed to demonstrate clinically meaningful effects in specific types of patients undergoing cardiac surgery.^8^

Taken together, while the risk prediction is undoubtedly important for clinical decision-making in individual patients, it should be considered as a model undergoing development that combines clinical judgment with new and established risk factors.
